# Supplementary material for: Serine metabolism remodeling after platinum-based chemotherapy identifies vulnerabilities in a subgroup of resistant ovarian cancers
Source: Nat Commun. 2022 Aug 5;13:4578. doi: 10.1038/s41467-022-32272-6 (PMC9355973; doi:10.1038/s41467-022-32272-6)
Supplement: Supplementary file 1 — Supplementary information [file 41467_2022_32272_MOESM1_ESM.pdf]

Supplementary information

Serine metabolism remodeling after platinum-based chemotherapy identifies vulnerabilities in a subgroup of resistant ovarian cancers

Van Nyen *et al.*, 2022

Supplementary Figure 1

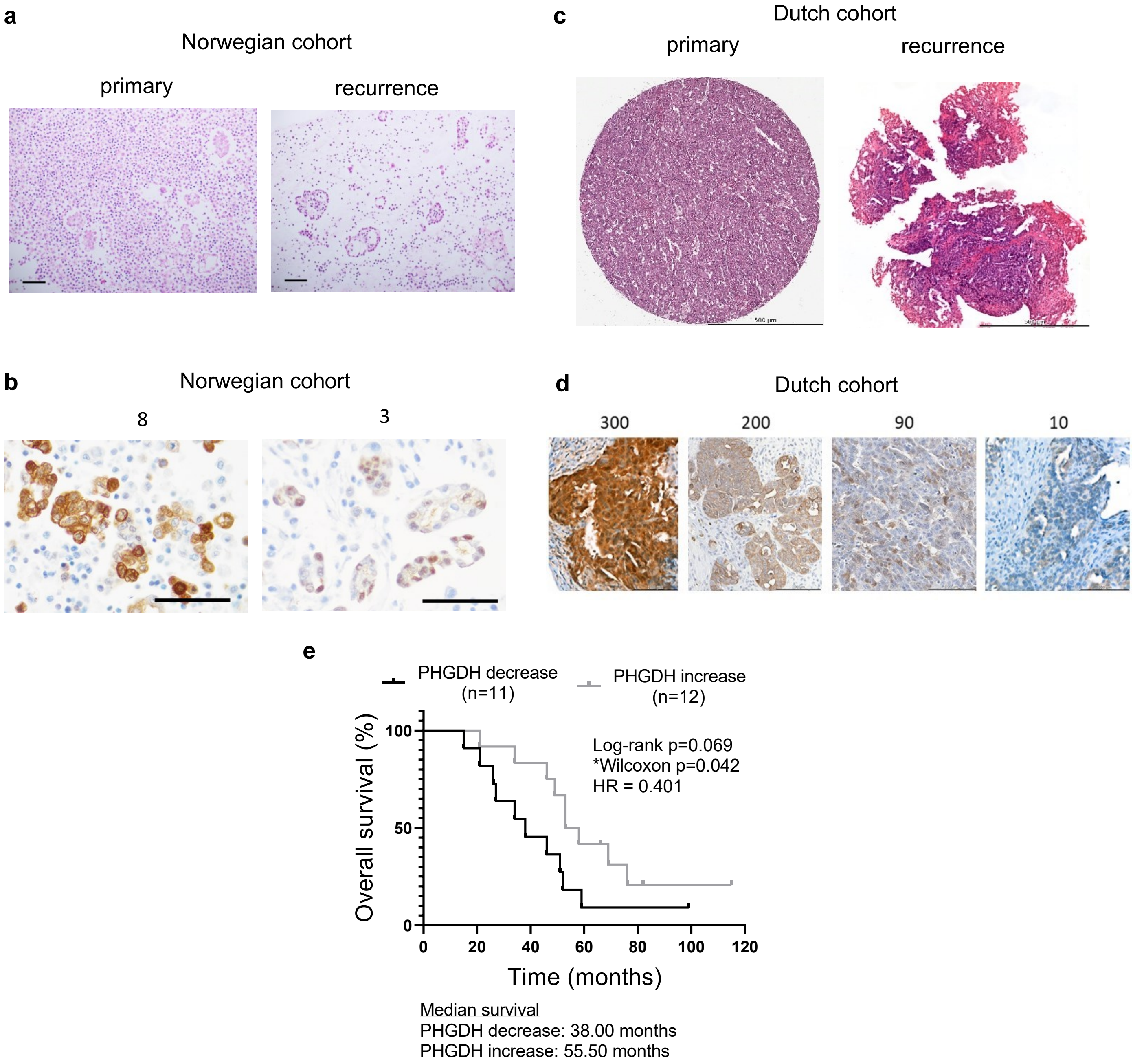

Supplementary Figure 1: A subset of tumor recurrences decrease PHGDH expression after platinum exposure. Related to Figure 1. (a) Representative H&E examples of primary and matched recurrences of pleural effusion samples of the Norwegian cohort, scale bar is 50 µm. (b) Representative scoring examples of the Norwegian cohort, scale bar is 50 µm. (c) Representative H&E examples of primary and matched tumor recurrences of the Dutch cohort, scale bar is 500 µm. (d) Representative scoring examples of the Dutch cohort, scale bar is 100 µm. (e) Overall survival of the PHGDH decreasing (n=11) and PHGDH increasing (n=12) subgroups of the Dutch patient cohort, Log-rank test two-tailed p=0.076, Gehan-Breslow-Wilcoxon test two-tailed p=0.042. Source data are provided as a Source Data file.

Supplementary Figure 2

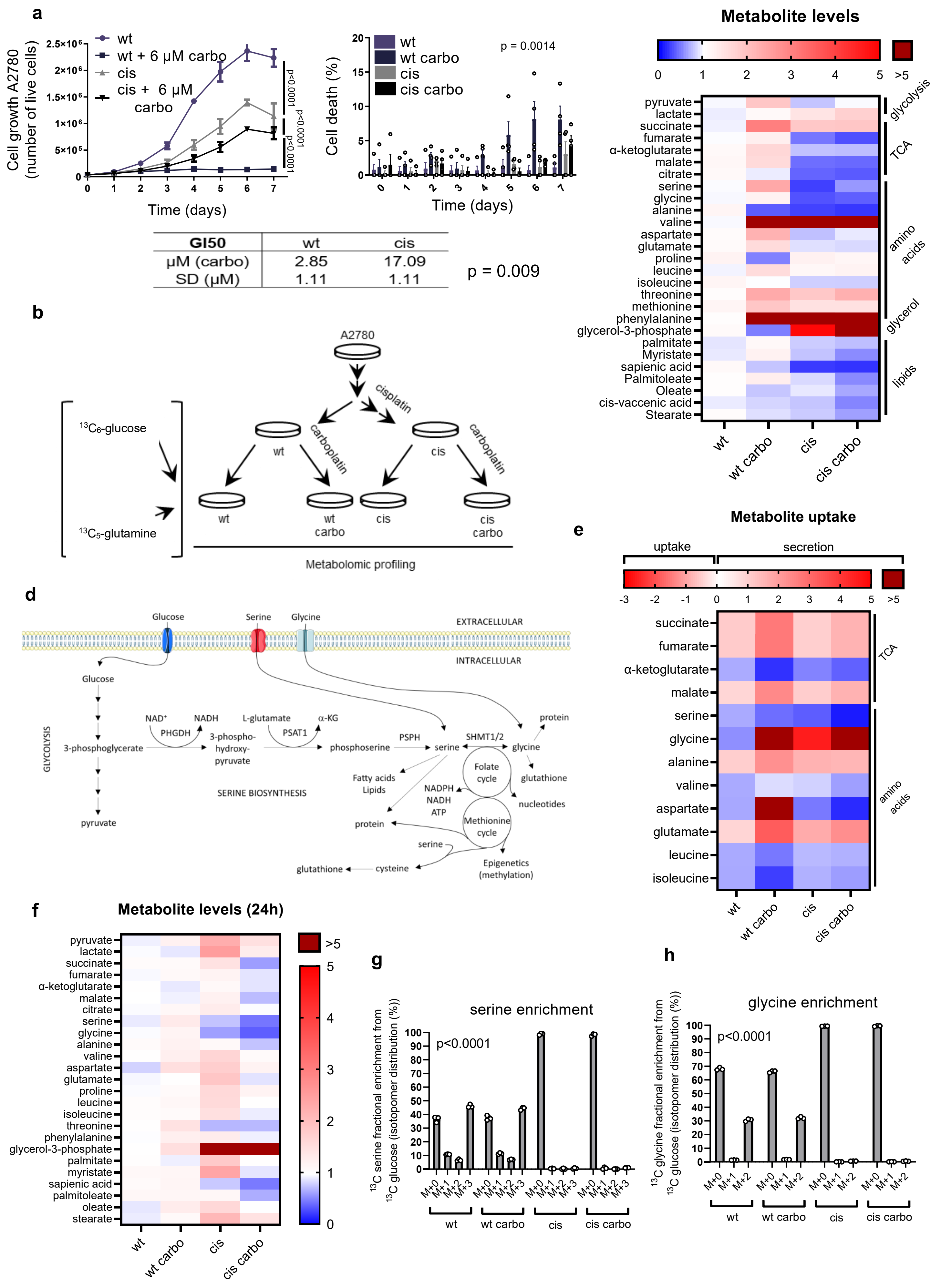

GI50	wt	cis
µM (carbo)	2.85	17.09
SD (µM)	1.11	1.11

Supplementary Figure 2: Ovarian cancer cells that develop resistance after platinum exposure adapt their metabolism and are characterized by lower serine biosynthetic activity. Related to Figure 2. (a) Growth and cell death of A2780wt and cis cells under 6 µM carboplatin and their carboplatin GI50 values determined by cell counting with trypan blue exclusion assay. Data are represented as mean ± SD, growth curve and cell death: n=4 biological replicates (with 3 technical replicates each), repeated measures two-way ANOVA with Tukey's multiple comparisons post-test, p<0.0001 for growth; GI50 n=3 biological replicates (with 3 technical replicates each), unpaired two-tailed t-test, p=0.009. (b) Schematic overview of experimental design. (c) Total metabolites levels in wt and cis cells, median values are plotted, representative figure with n=3 technical replicates and n=6 technical replicates for wt carbo (n=4 biological replicates). (d) Overview of serine biosynthetic pathway. (e) Metabolite uptake and secretion data of wt and cis cells, determined by GC-MS, median values are plotted, n=3 technical replicates. (f) Total metabolites levels in wt and cis cells after 24 hours, median values are plotted, n=3 technical replicates. (g-h) Serine (g) and glycine (h) isotopomer distribution of wt and cis cells grown with $^{13}C_6$-labeled glucose for 24 hours, n=3 technical replicates, Two-way ANOVA, data are represented as mean ± SD, p<0.0001. Some schematic art pieces in (d) were used and modified from Servier Medical Art. Servier Medical Art by Servier is licensed under a Creative Commons Attribution 3.0 Unported License (https://creativecommons.org/licenses/by/3.0/). Carbo = carboplatin treated cells, TCA = tricarboxylic acid cycle, αKG = α-ketoglutarate. Source data are provided as a Source Data file.

Supplementary Figure 3

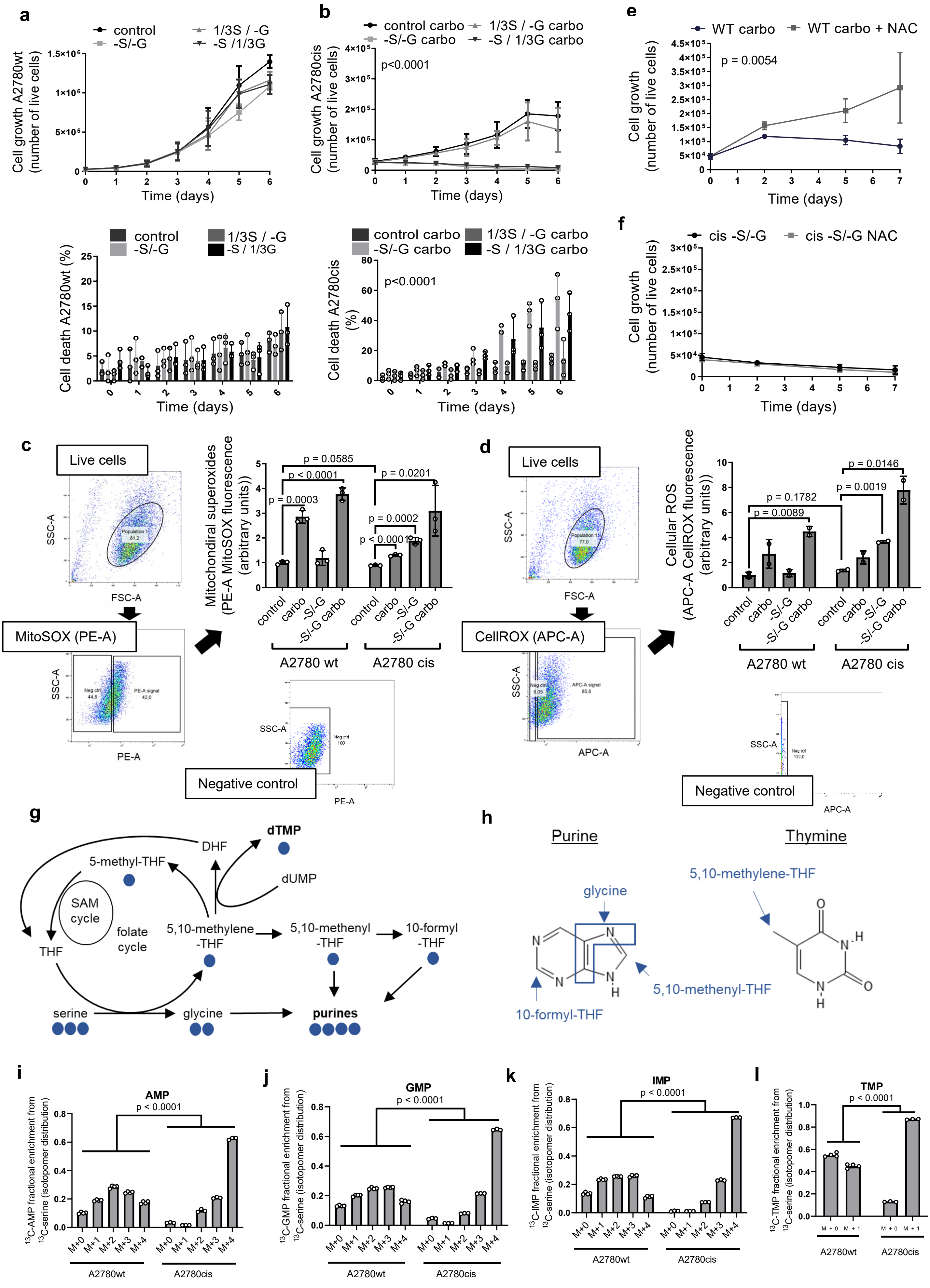

Supplementary Figure 3: Resistant cells rely on exogenous serine for nucleotides production. Related to figure 2. (a) Cell growth and cell death of wt cells under serine/glycine deprivation determined by trypan blue exclusion assay, n=3 biological replicates (with 3 technical replicates each), data are represented as mean ± SD. (b) Cell growth and cell death of cis cells treated with 6 µM carboplatin under serine/glycine deprivation determined by trypan blue exclusion assay, n=3 biological replicates (with 3 technical replicates each), Repeated measures two-way ANOVA, data are represented as mean ± SD, p<0.0001. (c-d) Mitochondrial superoxides determined by mean MitoSOX fluorescence (n=3 biological replicates with 3 technical replicates each) (c) and cytoplasmic ROS determined by mean CellROX fluorescence (n=2 biological replicates with 3 technical replicates each) (d) in wt and cis cells deprived from serine/glycine and under carboplatin treatment determined by flow cytometry at day 5, unpaired two-tailed t-tests between different conditions, data are represented as mean ± SD. Gating strategy is represented in figure. (e,f) Growth of wt cells under 6 µM carboplatin treatment and 200 µM NAC supplementation (n=3 biological replicates with 3 technical replicates each) (e) and growth of serine/glycine deprived cis cells with 200 µM NAC supplementation (n=2 biological replicates with 3 technical replicates each) (f), repeated measures two-way ANOVA, data are represented as mean ± SD, p=0.0054 for (e). (g,h) Schematic overview of serine contribution to purines and thymine. (i-k) isotopomer distribution of purines (i: AMP, j:GMP, k: IMP) after feeding cells with $^{13}C_3$-serine for 5 days, n=4 technical replicates for wt and n=3 technical replicates for cis cells, two-way ANOVA with Sidak's multiple comparisons post-test, data are represented as mean ± SD, p<0.0001 for all. (l) isotopomer distribution of TMP after feeding cells with $^{13}C_3$-serine for 5 days n=4 technical replicates for wt and n=3 technical replicates for cis cells, two-way ANOVA with Sidak's multiple comparisons post-test, data are represented as mean ± SD, p<0.0001. Chemical structures in (h) are made using PubChem Sketcher V2.4. S = serine, G = glycine, carbo = carboplatin treated, NAC = N-acetylcysteine, THF = tetrahydrofolate, DHF = dihydrofolate, SAM = S-adenosylmethionine. Source data are provided as a Source Data file.

Supplementary Figure 4

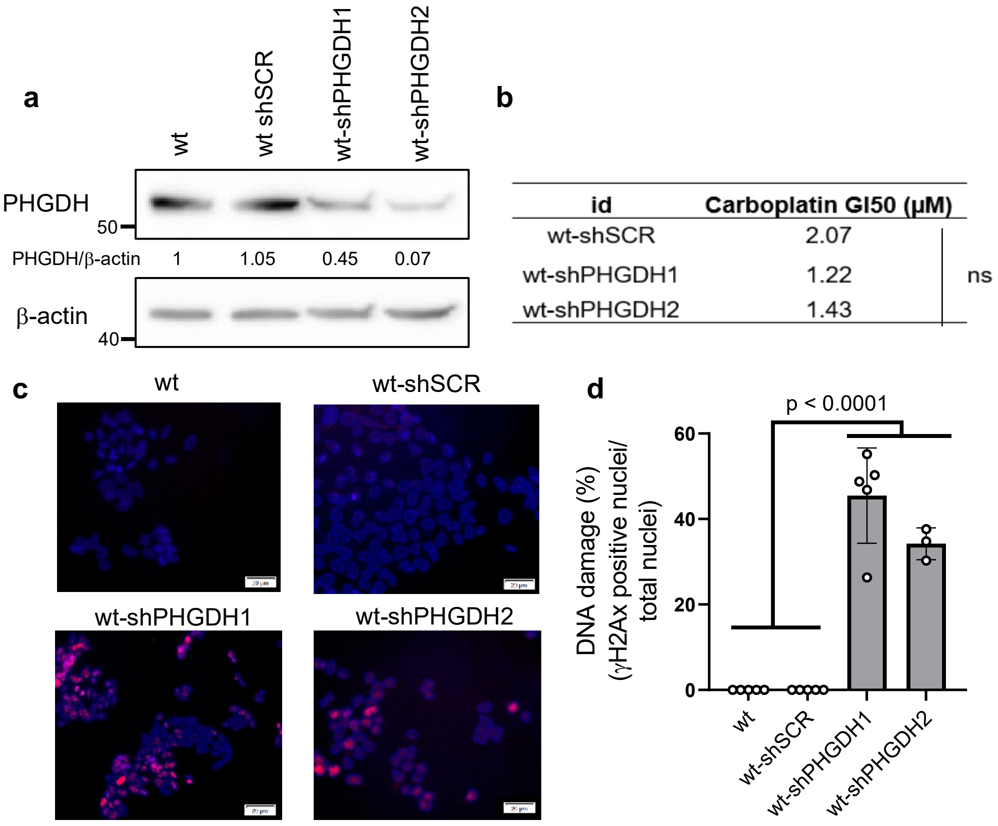

Supplementary Figure 4: Chemo-naïve A2780 cells are highly dependent on PHGDH. Related to figure 3. (a) Representative western blot of PHGDH knock-down in platinum sensitive A2780wt cells, using two different shPHGDHs, n=2 biological replicates. (b) carboplatin GI50s of PHGDH knocked down A2780wt cells, n=3 biological replicates (with 3 technical replicates each), unpaired two-failed t-tests. (c, d) Representative images of γH2Ax staining (red) and DAPI (blue) determined by immunofluorescence and fluorescence microscopy, scale bar is 20 μm (c) and quantification of γH2Ax positive nuclei using ImageJ cell counting tool (d), n=5 technical replicates except for wt-shPHGDH2 n=3 technical replicates, ordinary one-way ANOVA with multiple comparison Tukey's post-test, data are represented as mean ± SD, $p<0.0001$. SCR=scramble, ns=not significant. Source data are provided as a Source Data file.

Supplementary Figure 5

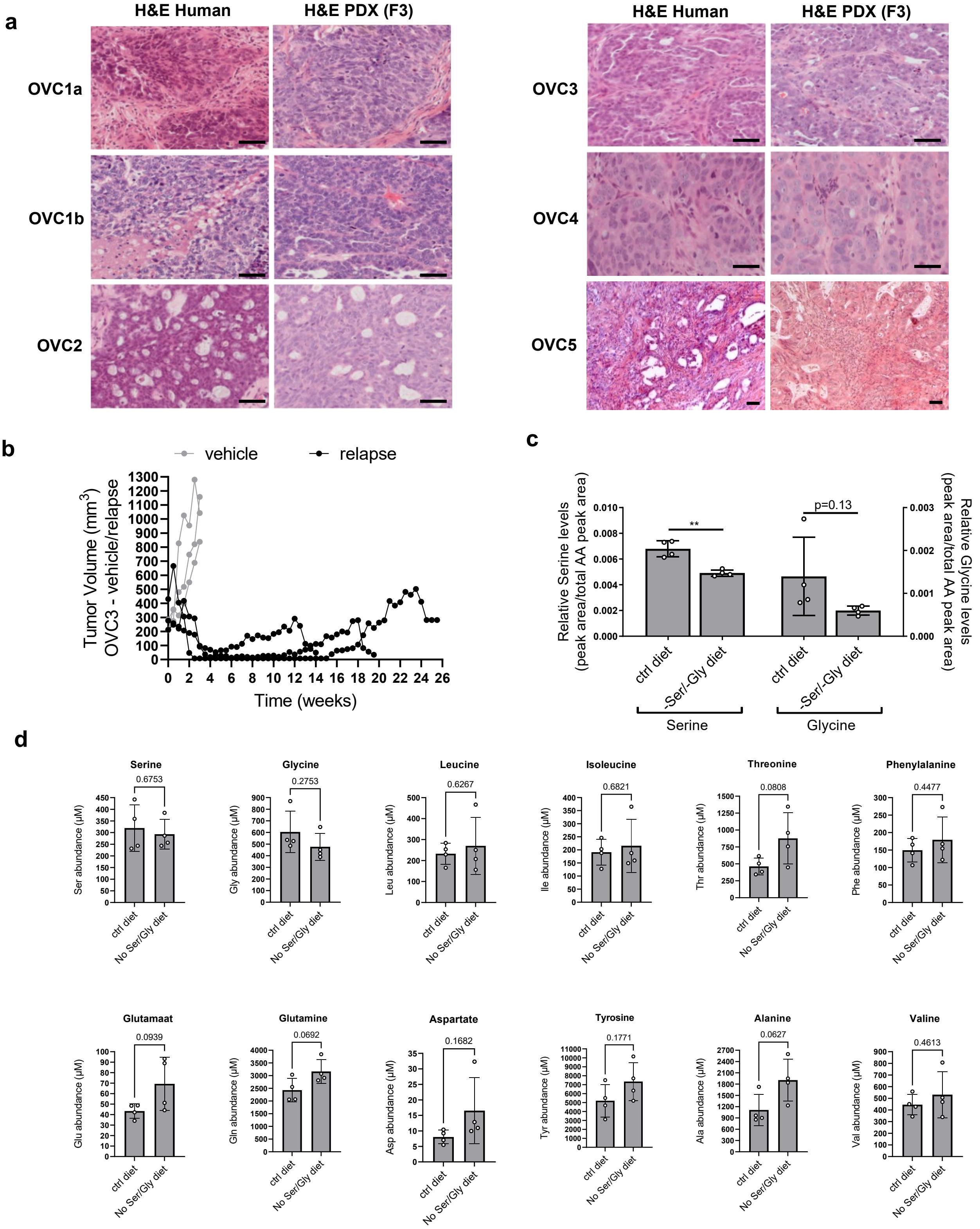

Supplementary Figure 5: Characterization of intratumor and plasma amino acid levels of PDX mice under serine/glycine free diet. Related to Figure 4. (a) H&E stainings of the used PDX models and their matched original patients, n=1 slide per tumor tissue, n>3 mice per condition and n>3 microscopic fields per slide, scale bar is 100 µm. (b) tumor growth in individual mice of OVC3, vehicle treated (n=3) and carboplatin treated - relapsed – carboplatin treated (n=3). (c) relative plasma serine and glycine levels in control mice with control diet or with serine/glycine free diet, n=4 mice for each condition (quantification was done 2 times), unpaired two-tailed t-tests, data are represented as mean ± SD. (d) Total plasma amino acid abundance in control mice with control diet or with serine/glycine free diet, n=4 mice for each condition (quantification was done 2 times), unpaired two-tailed t-tests, data are represented as mean ± SD. Source data are provided as a Source Data file.

Supplementary Figure 6

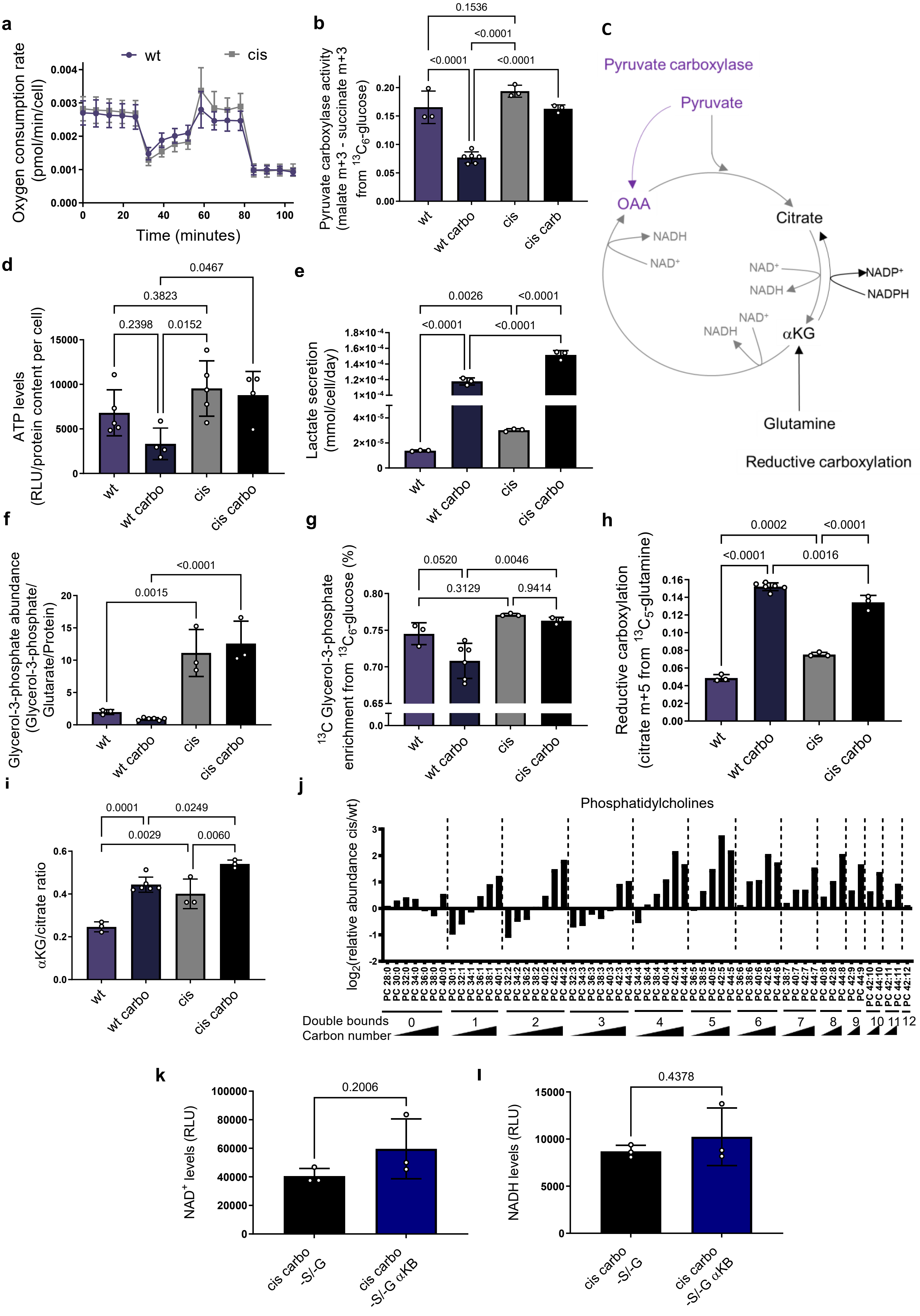

Supplementary Figure 6: Platinum-resistant cells reshuffle their metabolism towards a NAD$^+$-regenerating phenotype. Related to figure 5. (a) Oxygen consumption rate determined with an Agilent Seahorse XF Analyzer, n=7 technical replicates, data are represented as mean ± SD. (b) Pyruvate carboxylase activity determined as malate m+3 levels – succinate m+3 levels from $^{13}C_6$-glucose determined by GC-MS, representative figure with n=3 technical replicates (n=2 biological replicates), ordinary one-way ANOVA with Tukey's multiple comparison post-test, data are represented as mean ± SD, p<0.0001. (c) Schematic overview of pyruvate carboxylation and reductive carboxylation of glutamine. (d) ATP levels determined with ATP-lite Promega bioluminescent assay, n=5 biological replicates (with 3 technical replicates each), ordinary one-way ANOVA with Tukey's multiple comparison post-test, data are represented as mean ± SD, p=0.0170. (e) Lactate secretion determined by LC-MS, n=3 technical replicates, ordinary one-way ANOVA with Tukey's multiple comparison post-test, data are represented as mean ± SD, p<0.0001. (f) Glycerol-3-phosphate levels determined by GC-MS, representative figures with n=3 technical replicates (n=3 biological replicates), ordinary one-way ANOVA with Tukey's multiple comparison post-test, data are represented as mean ± SD, p<0.0001. (g) Glycerol-3-phosphate fractional enrichment from $^{13}C_6$-glucose determined by GC-MS, representative figure with n=3 technical replicates (n=2 biological replicates), ordinary one-way ANOVA with Tukey's multiple comparison post-test, data are represented as mean ± SD, p=0.0010. (h) Reductive carboxylation determined by citrate m+5 from $^{13}C_5$-glutamine, representative figure with n=3 technical replicates (n=2 biological replicates), ordinary one-way ANOVA with Tukey's multiple comparison post-test, data are represented as mean ± SD, p<0.0001. (i) α-ketoglutarate (αKG)/citrate ratio, αKG and citrate both determined separately by GC-MS, representative figure with n=3 technical replicates (n=2 biological replicates), ordinary one-way ANOVA with Tukey's multiple comparison post-test, data are represented as mean ± SD, p<0.0001. (b, e-i) representative figures with n=3 technical replicates for all, except n=6 technical replicates for wt carbo. (j) Lipid profile of phosphatidylcholines in cis cells relatively to wt cells, n=3 technical replicates, mean is plotted. (k-l) NAD$^+$ levels (k) and NADH levels (l) determined by NAD$^+$/NADH-Glo bioluminescent Promega assay, n=3 biological replicates (with 3 technical replicates each), unpaired two-tailed t-test, data are represented as mean ± SD, p=0,2006 for (k) and p=0.4378 for (l). OAA = oxaloacetic acid, αKG = α-ketoglutarate, αKB = α-ketobutyrate, PC = phosphatidylcholine, S = serine, G = glycine. Source data are provided as a Source Data file.

Supplementary Figure 7

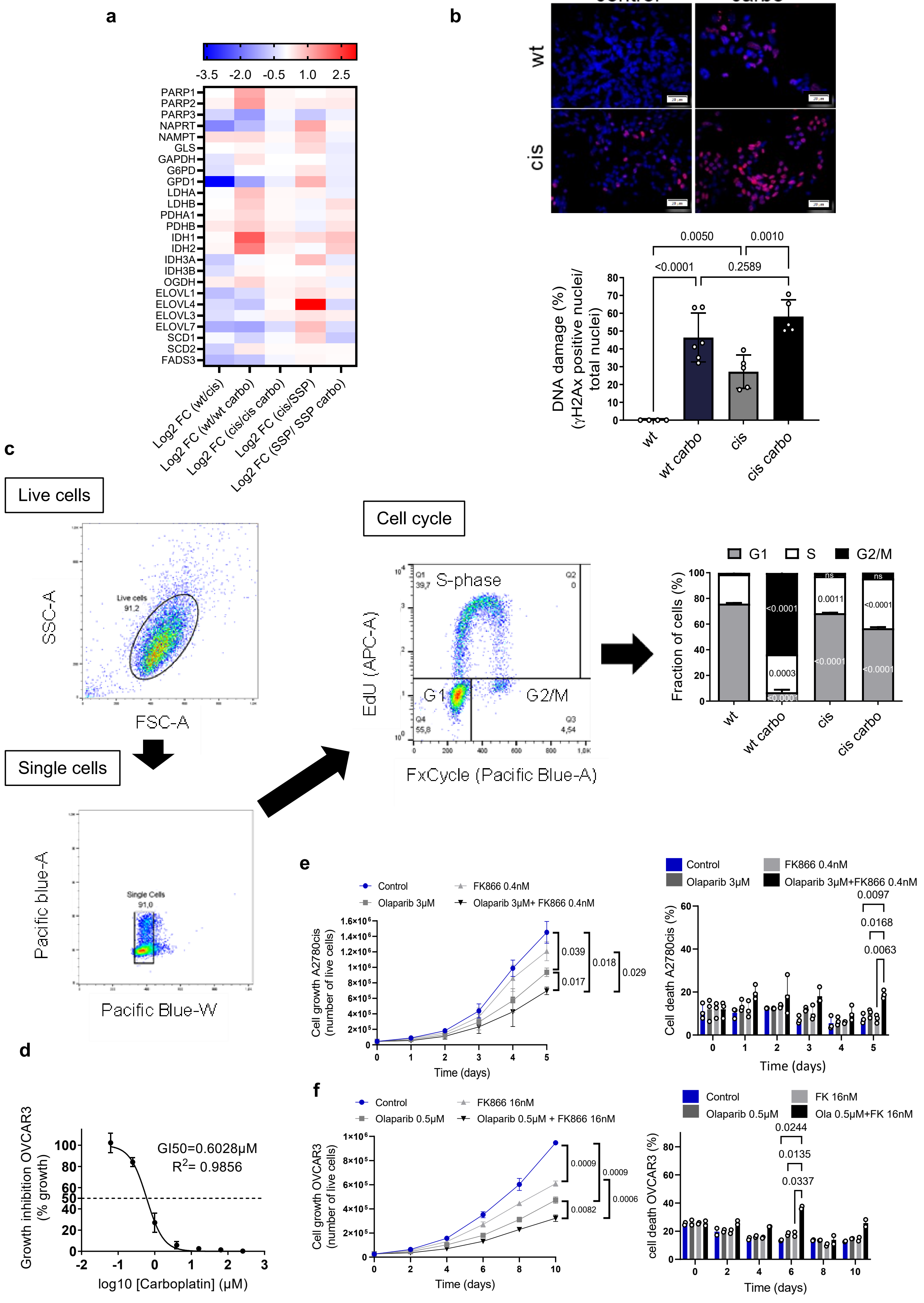

Supplementary Figure 7: Platinum-resistant cells have high DNA damage tolerance and PARP inhibition has a synergistic effect with platinum treatment. Related to Figure 6. (a) Analysis of main NAD^+-related enzymes using RNA-seq data of wt, cis and SSP cells, mean values are plotted, n=3 technical replicates. (b) Representative images of γH2Ax staining (red) and DAPI (blue) determined by immunofluorescence and fluorescence microscopy, scale bar is 20 µm (upper) and quantification of γH2Ax positive nuclei using ImageJ cell counting tool (lower), representative figure with wt n=4, wt carbo n=6, cis (±carbo) n=5 technical replicates (staining was performed on 3 different slides per condition each time and staining was performed as n=2 independent biological replicates) , ordinary one-way ANOVA with multiple comparison Tukey's post-test, data are represented as mean ± SD, $p<0.0001$. (c) Cell cycle analysis of wt and cis cells using EdU and FxCycle fluorescent staining followed by flow cytometry, representative graph with n=3 technical replicates (n=3 biological replicates), two-way ANOVA with Tukey's multiple comparison post-test each compared to wt cells, error bars represent SEM, $p<0.0001$. Gating strategy is indicated in the figure. (d) Carboplatin GI50 value of OVCAR3 cells, n=3 biological replicates (with 3 technical replicates each), unpaired two-tailed-test, data are represented as mean ± SD. (e,f) Growth and cell death of A2780 cis cells (e) and OVCAR3 cells (f) treated with olaparib, FK866 or its combination. Mean values ± SD are plotted, n=3 biological replicates (with 3 technical replicates each) for all except for cell death in (f) where n=2 biological replicates (with 3 technical replicates each), two-way ANOVA with Tukey's multiple comparison post-test. $p<0.0001$ for growth and $p=0.2990$ for cell death in (e); $p<0.0001$ for both growth and cell death in (f). Carbo = carboplatin treated cells. Source data are provided as a Source Data file.

Supplementary Figure 8

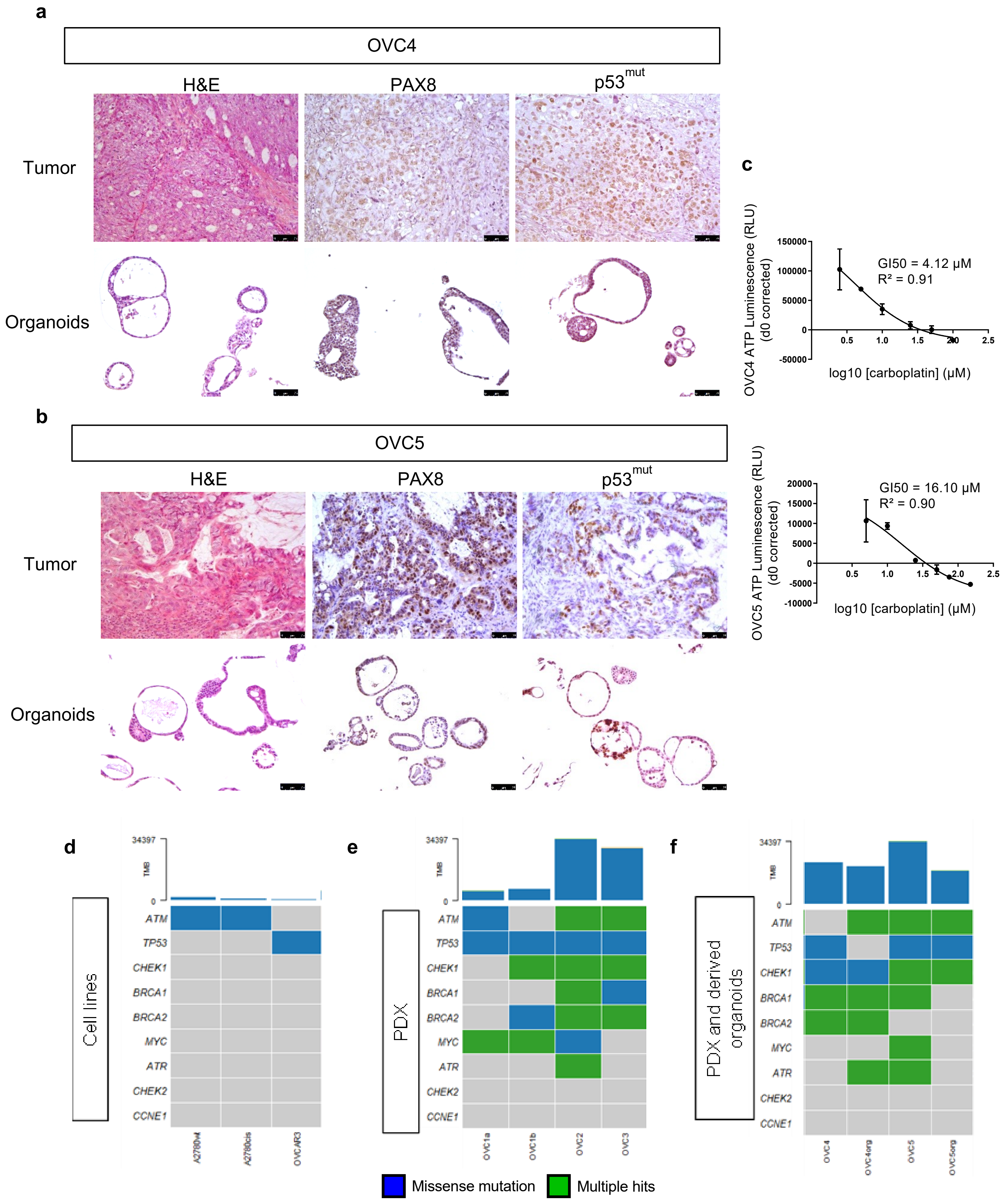

Supplementary Figure 8: Histological and genetic characterization of OVC4 and OVC5 PDX-derived organoids. Related to Figure 7. (a,b) Characterization of the platinum resistant ovarian cancer model OVC4 (a) and the mucinous ovarian cancer model OVC5 (b) organoids by H&E, PAX8 and mutant p53 IHC and their matched PDX tumors, scale bar = 75 µm, n=2 slides were stained for each condition. (c) Carboplatin GI50 values of OVC4 (upper) and OVC5 (lower) determined by ATP measurements after 5 days of treatment and corrected for day 0 measurements, n=3 biological replicates (with 6 technical replicates each), mean values ± SD are plotted. (d-f) Mutational analysis of main homologous recombination related genes of the cell lines (d), PDX models (e) and organoids with matched PDX models (f). Source data are provided as a Source Data file.

Supplementary Table 1

Supplementary Table 1: Clinical data and PHGDH scoring of matched primary biopsies samples and late stage recurrent samples of pleural effusions from the Norwegian cohort. HGSC = high-grade serous ovarian carcinoma, OS=overall survival.

Pair	Age	Histology	FIGO	PHGDH score primary sample	Response to 1st line platinum	Time to recurrent biopsy (months)	PHGDH score recurrent biopsy	PHGDH score difference (primary–recurrence)	PHGDH score difference (%)	OS (months)
1	40-50	HGSC	IV	6	partial	30	6	0	0	32
2	60-70	HGSC	III	6	complete	68	8	+2	+33	72
3	60-70	HGSC	IV	6	complete	14	4	-2	-33	18
4	50-60	HGSC	III	8	complete	11	8	0	0	26
5	70-80	HGSC	III	8	partial	13	3	-5	-63	17
6	70-80	HGSC	IV	8	partial	10	4	-4	-50	14
7	50-60	HGSC	IV	8	complete	11	3	-5	-63	17
8	60-70	HGSC	III	3	complete	41	2	-1	-33	48
9	60-70	HGSC	III	8	complete	17	6	-2	-25	24

Supplementary Table 2

Supplementary Table 2: Clinical data and PHGDH scoring of matched primary and recurrent tumor biopsies of the Dutch cohort. PDS=primary debulking surgery, NACT= neo-adjuvant chemotherapy, OS= overall survival, NA = not available.

Pair	Age	First line chemo	NACT or PDS	FIGO stage	Metastasis	PHGDH score primary sample	Interval primary-recurrent biopsy (months)	PHGDH score recurrent biopsy	PHGDH score difference (primary–recurrent biopsy)	PHGDH score difference (%)	OS (months)
1	50-60	carboplatin-paclitaxel	NACT	III	No	200	39	10	-190	-95	46
2	70-80	paclitaxel-carboplatin	NACT	IV	Lymph nodes	180	15	120	-60	-33	27
3	60-70	carboplatin-paclitaxel	NACT	IV	Visceral	90	72	120	30	33	115
4	60-70	carboplatin-paclitaxel	NACT	III	No	140	19	240	100	71	69
5	60-70	carboplatin-paclitaxel	NACT	III	No	200	9	270	70	35	34
6	60-70	carboplatin-paclitaxel	NACT	III	No	200	4	80	-120	-60	34
7	60-70	carboplatin-paclitaxel	NACT	III	No	300	4	270	-30	-10	38
8	60-70	carboplatin-paclitaxel	NACT	III	No	160	28	120	-40	-25	99
9	60-70	carboplatin-paclitaxel	PDS	NA	No	180	56	270	90	50	76
10	70-80	carboplatin-paclitaxel	PDS	III	No	200	38	270	70	35	46
11	60-70	carboplatin-paclitaxel	NACT	IV	Visceral	270	9	180	-90	-33	15
12	50-60	other chemo	PDS	III	No	240	49	140	-100	-42	59
13	50-60	carboplatin-paclitaxel	NACT	IV	Pleural	200	22	270	70	35	53
14	70-80	carboplatin-paclitaxel	NACT	III	No	180	31	270	90	50	58
15	50-60	carboplatin-paclitaxel	PDS	III	No	60	28	140	80	133	82
16	60-70	carboplatin-paclitaxel	NACT	III	No	160	40	270	110	69	53
17	70-80	carboplatin-paclitaxel	NACT	III	No	210	17	160	-50	-24	52
18	70-80	carboplatin-paclitaxel	NACT	III	No	270	6	300	30	11	21
19	50-60	carboplatin-paclitaxel	PDS	III	No	300	18	270	-30	-10	26
20	70-80	carboplatin-paclitaxel	NACT	III	No	270	18	270	0	0	41
21	70-80	carboplatin-paclitaxel	NACT	IV	Pleural	270	4	270	0	0	11
22	40-50	carboplatin-paclitaxel	NACT	IV	Other	120	21	160	40	33	49
23	40-50	carboplatin-paclitaxel	NACT	III	No	240	15	10	-230	-96	51
24	50-60	carboplatin-paclitaxel	PDS	III	No	180	33	240	60	33	66
25	50-60	carboplatin-paclitaxel	NACT	IV	Lymph nodes	200	15	60	-140	-70	21

Supplementary Table 3

Supplementary Table 3: Overview of the different PDX models and derived organoids used, with patients' clinical data and reported response to carboplatin in mice. NA = not available, carbo = carboplatin.

Tumor ID	FIGO stage	Primary/ recurrence	Platinum exposure	Response in patient	Platinum status for the patient	Response in PDX
OVC1a	3b	Primary	Chemonaive	Complete response	Sensitive	Tumor regression
OVC1b[a]	/	Recurrence	1 line	Complete response	Sensitive	Tumor regression
OVC2	1c	Recurrence	4 lines	Progression under carbo	Resistant	Progression under carbo
OVC3	3c	Primary	Chemonaive	Complete response but relapse after 5 months	Resistant	Tumor regression after first line Mixed response to second line
OVC4	1a	Recurrence	4 lines	Progression under carbo	Resistant	Progression under carbo
OVC5[b]	NA	Recurrence	1 line	Progression under carbo	Resistant	/

[a]OVC1b is established from relapse of OVC1a, 13 monthes after the last chemo dose
[b]OVC5 is derived from a mucinous ovarian cancer tumor
