# Supplementary material for: Serine metabolism remodeling after platinum-based chemotherapy identifies vulnerabilities in a subgroup of resistant ovarian cancers
Source: Nat Commun. 2022 Aug 5;13:4578. doi: 10.1038/s41467-022-32272-6 (PMC9355973; doi:10.1038/s41467-022-32272-6)
Supplement: Supplementary file 3 — Reporting Summary [file 41467_2022_32272_MOESM3_ESM.pdf]

nature research

Corresponding author(s): Frédéric Amant, Daniela Annibali

Last updated by author(s): May 25, 2022

Reporting Summary

Nature Research wishes to improve the reproducibility of the work that we publish. This form provides structure for consistency and transparency in reporting. For further information on Nature Research policies, see our Editorial Policies and the Editorial Policy Checklist.

Statistics

For all statistical analyses, confirm that the following items are present in the figure legend, table legend, main text, or Methods section.

n/a	Confirmed	
☐	☒	The exact sample size (*n*) for each experimental group/condition, given as a discrete number and unit of measurement
☐	☒	A statement on whether measurements were taken from distinct samples or whether the same sample was measured repeatedly
☐	☒	The statistical test(s) used AND whether they are one- or two-sided<br>*Only common tests should be described solely by name; describe more complex techniques in the Methods section.*
☒	☐	A description of all covariates tested
☐	☒	A description of any assumptions or corrections, such as tests of normality and adjustment for multiple comparisons
☐	☒	A full description of the statistical parameters including central tendency (e.g. means) or other basic estimates (e.g. regression coefficient) AND variation (e.g. standard deviation) or associated estimates of uncertainty (e.g. confidence intervals)
☐	☒	For null hypothesis testing, the test statistic (e.g. *F*, *t*, *r*) with confidence intervals, effect sizes, degrees of freedom and *P* value noted<br>*Give P values as exact values whenever suitable.*
☒	☐	For Bayesian analysis, information on the choice of priors and Markov chain Monte Carlo settings
☒	☐	For hierarchical and complex designs, identification of the appropriate level for tests and full reporting of outcomes
☐	☒	Estimates of effect sizes (e.g. Cohen's *d*, Pearson's *r*), indicating how they were calculated

Our web collection on statistics for biologists contains articles on many of the points above.

Software and code

Policy information about availability of computer code

Data collection

Light microscopy pictures of cells: Leica Application Suite Version 3.7.0 imaging software
Light microscopy pictures of tissue: LAS AF imaging software
Fluorescence microscopy pictures: cellSens Dimension software
WB capturing: cSeries Capture Software (Azure c600)
FACS data collection: BD FACS Diva software
Bioluminiscent assays data collection: Perkin Elmer Working Station
Spectrophotometric data collection: SkanIt for Multiskan FC 3.1
LC-MS data collection: Thermo Xcalibur (v4.0)
GC-MS data collection: Agilent Masshunter (version 10.1 build 10.1.48)
No other specific software were used for data collection.

Data analysis

Data representation: GraphPad Prism 9
Data analysis: Excel 2019 and GraphPad Prism 9
Flow cytometry data analysis: FlowJo 10.7.1
Quantification of WB: ImageJ
Metabolomics analysis: Masshunter version 10.0, Matlab R2016b, Excel 2019
RNAseq analysis: Raw RNAseq FASTQ files were aligned with STAR (version 020201) to the human genome GRCh37.p13. The file formats were then converted using SAMtools (version 0.1.19-96b5f2294a). Reads were counted at exons of protein-coding genes using HTSeq (version 0.5.4p3) with gencode.v19 annotation. Normalization of library size for the read counts and differential expression analysis was performed using DEseq and DEseq2 with standard parameters.
WES analysis: WES were processed using an in-house pipeline. Base-calling was performed with bcl2fastq (version 2.19.0). Reads were aligned to the human reference genome GRCh38 using BWA mem (version 0.7.17), duplicate reads were marked using Picard MarkDuplicates (version 2.22.1) and base quality scores were recalibrated using GATK BaseRecalibrator and ApplyBQSR (version 4.1.7). Finally Single Nucleotide Variants (SNVs) and small indels were called using GATK HaplotypeCaller (version 4.1.7) and annotated using Annovar (version 2019Oct24).

Variant filtering was performed in R based on quality score, coverage and pathogenicity score. Variants with a Phred corrected quality score of <50, a coverage of <10x and >243 and Phred corrected CADD pathogenicity score <15 were removed. Variants that met these requirements were incorporated into the final mutation annotation format (MAF) and further processed with the R package maftools for visualization."

For manuscripts utilizing custom algorithms or software that are central to the research but not yet described in published literature, software must be made available to editors and reviewers. We strongly encourage code deposition in a community repository (e.g. GitHub). See the Nature Research guidelines for submitting code & software for further information.

Data

Policy information about availability of data

All manuscripts must include a data availability statement. This statement should provide the following information, where applicable:

- Accession codes, unique identifiers, or web links for publicly available datasets
- A list of figures that have associated raw data
- A description of any restrictions on data availability

Data from the TCGA were obtained using cBioPortal: https://www.cbioportal.org/ (Ovarian Cystadenocarincoma, TCGA, Nature 2011). Gene expression data from the CCLE were obtained using cBioPortal: https://www.cbioportal.org/ (Cancer Cell Line Encyclopedia, Broad 2019). Drug sensitivity data were obtained from the GDSC, using https://www.cancerrxgene.org/ (STUDY: olaparib, Pubchem 23725625, sample size 762, screening site Sanger, dataset GDSC2). These data were accessed on Nov 2, 2020.

RNA-seq data have been deposited into the NCBI Gene Expression Omnibus (GEO) database (http://www.ncbi.nlm.nih.gov/geo/) under accession number GSE176218.

WES data have been deposited into the SRA database (https://www.ncbi.nlm.nih.gov/sra) under accession number PRJNA816435.

Data represented in the figures are available in Excel as source data files named "Source data main figures", "Source data Supplementary Figures" and "Source data western blots". All other additional and raw data supporting the findings of this study are available from the corresponding author on reasonable request.

Field-specific reporting

Please select the one below that is the best fit for your research. If you are not sure, read the appropriate sections before making your selection.

☒ Life sciences ☐ Behavioural & social sciences ☐ Ecological, evolutionary & environmental sciences

For a reference copy of the document with all sections, see nature.com/documents/nr-reporting-summary-flat.pdf

Life sciences study design

All studies must disclose on these points even when the disclosure is negative.

Sample size

No statistical method was used to predetermine sample size. Sample sizes were chosen based on commonly adopted standards, resulting in statistically meaningful comparisons.

In vitro experiments were performed at least 3 times with 3 replicates in each experiment, except if stated otherwise.

For animal experiments, enough animals per treatment group were used for statistical comparison, and numbers were determined based on previous experience. Overall, around ~10 mice were included per treatment group (exact numbers indicated in figure legends), being in line with other reports that study amino acid dependencies and therapy response in PDX models (e.g.: https://doi.org/10.1038/s41586-019-1437-3)

For patient sample inclusion, all samples with primary and matched recurrence biopsies that we were able to obtain were included.

Data exclusions

No data were excluded.

Replication

The number of independent mice/samples is mentioned in each figure/figure legend. In vitro experiments were at least performed 3 times with 3 replicates in each experiment, except is stated otherwise. All attempts at replication were successful

For patient IHC scoring, different expert pathologists scored the different sample cohorts.
For the Norwegian cohort, IHC was performed once and scored by a pathologist (BD).
For the Dutch cohort, 2-4 cores (with an average of 3 cores) per patient of the primary tumors were incorporated in a TMA while for recurrences 1 whole-slide staining was available per patient (due to limited tissue availability). These slides were scored by different pathologists then the Norwegian cohort. Primary stainings of the Dutch cohort were scored by 2 different pathologists (HMH + additional pathologist at CFMP NKI) and recurrences were scored by HMH and LvW.

Randomization

Mice were randomly assigned to a treatment group as soon as they were ready to be included. Different treatment groups were processed identically and animals in the same treatment groups were exposed to the same environment.

Microscopic fields of cells was randomly determined at least 3 times per condition and a representative picture is shown. Microscopic fields of patient's IHC was determined based on the presence of sufficient tumor cells and average staining levels across different fields.

No other randomization was needed in this study.

Blinding

Experiments were performed by the same researcher(s) and experiments were thus not blinded during data collection.
However, since mice were randomly allocated in treatment cohorts (and different treatments were mixed in the cages), measurements of tumor volumes was unbiased (not knowing all the treatment arms at moments of measurements).
No other blinding was needed in this study.

Reporting for specific materials, systems and methods

We require information from authors about some types of materials, experimental systems and methods used in many studies. Here, indicate whether each material, system or method listed is relevant to your study. If you are not sure if a list item applies to your research, read the appropriate section before selecting a response.

Materials & experimental systems

n/a	Involved in the study
☐	☒ Antibodies
☐	☒ Eukaryotic cell lines
☒	☐ Palaeontology and archaeology
☐	☒ Animals and other organisms
☐	☒ Human research participants
☒	☐ Clinical data
☒	☐ Dual use research of concern

Methods

n/a	Involved in the study
☒	☐ ChIP-seq
☐	☒ Flow cytometry
☒	☐ MRI-based neuroimaging

Antibodies

Antibodies used

anti-PHGDH (HPA021241, 1:2000 for WB, 1/4000 for Norway IHC, 1/8000 for Dutch IHC, Sigma), anti-PSPH (HPA020376, 1:1000 for WB, Sigma), anti-PSAT1 (CPTC-PSAT1-2, 1:500, Developmental Studies Hybridoma Bank), anti-PARP1 (AFFN-PARP1-17B10, 1:150, Developmental Studies Hybdridoma Bank), anti-gH2Ax (Ser139) (20E3, 1:600, #9718, Cell Signaling Technology), anti-b-actin (13E5, 1:5000, #4970, Cell Signaling Technology), anti-ATF4 (D4B8, 1:1000, #11815, Cell Signaling Technology), Mouse anti-p53 Monoclonal Antibody [DO-1] - ChIP grade (ab1101, 1:200 for IHC, Abcam), PAX8 Polyclonal antibody (10336-1-AP, 1:1200 for IHC, Proteintech), secondary peroxidase conjugated goat anti-rabbit (111-035-003, 1:5000 for wb and 1:10 000 for actin wb, Jackson ImmunoResearch), goat anti-mouse (115-035-044, 1:10 000, Jackson ImmunoResearch), secondary donkey anti-rabbit Alexa Fluor 594 (1:800, Invitrogen A-21207, Life Technologies)

Validation

Except for anti-PSAT1 and anti-PARP1 anbidodies, all antibodies were obtained from commercial sources and validated according to the manufcturers' instruction.
- rabbit anti-PHGDH was validated for IHC, WB and ICC-IF in human, mouse and rat and validation was done by Atlas Antibodies - Human Protein Atlas: www.atlasantibodies.com/products/HPA021241.
- rabbit anti-PSPH was validated for IHC and WB in human samples and validation was done by Atlas Antibodies - Human Protein Atlas: www.atlasantibodies.com/products/HPA020376.
- rabbit anti-gH2Ax was validated for WB, IHC, IF and flow cytometry in human, mouse, rat and monkey. Validation was done by Cell Signaling Technologies: https://www.cellsignal.com/products/primary-antibodies/phospho-histone-h2a-x-ser139-20e3-rabbit-mab/9718
- rabbit anti-b-actin was validated for WB, IHC-P, IF-IC and flow cytometry in human, mouse, rat, monkey, bovine and pig samples. Validation was done by Cell Signaling Technologies: https://www.cellsignal.com/products/primary-antibodies/b-actin-13e5-rabbit-mab/4970.
- rabbit anti-ATF4 was validated for WB, IP, IF and ChIP in human, mouse and rat samples. Validation was done by Cell Signaling Technologies: https://www.cellsignal.com/products/primary-antibodies/atf-4-d4b8-rabbit-mab/11815
- mouse anti-p53 was validated for Flow Cytometry, ICC, ChIP, IHC-P, WB cytometry in human samples. Validation was done by Abcam: https://www.abcam.com/p53-antibody-do-1-chip-grade-ab1101.html .
- rabbit anti-PAX8 was validated for WB, IHC, IF, ELISA and flow cytometry in human, mouse and rat samples. Validation was done by Proteintech: https://www.ptglab.com/products/PAX8-Antibody-10336-1-AP.htm#publications
- secondary peroxidase conjugated goat anti-rabbit: Based on immunoelectrophoresis and/or ELISA, the antibody reacts with whole molecule rabbit IgG. It also reacts with the light chains of other rabbit immunoglobulins. No antibody was detected against non-immunoglobulin serum proteins. The antibody may cross-react with immunoglobulins from other species. The whole IgG form of antibodies is suitable for the majority of immunodetection procedures and is the most cost effective. https://www.jacksonimmuno.com/catalog/products/111-035-003.
- secondary peroxidase conjugated goat anti-mouse: Based on immunoelectrophoresis and/or ELISA, the antibody reacts with both mouse IgG and IgM. It also reacts with the light chains of other mouse immunoglobulins. No antibody was detected against non-immunoglobulin serum proteins. The antibody may cross-react with immunoglobulins from other species. The whole IgG form of antibodies is suitable for the majority of immunodetection procedures and is the most cost effective. https://www.jacksonimmuno.com/catalog/products/115-035-044.
- secondary donkey anti-rabbit alexa fluor 594 was validated for IHC, ICC/IF and flow cytometry and reacts with rabbit Gamma Immunoglobins Heavy and Light chains. To minimize cross-reactivity, these donkey anti-rabbit IgG whole antibodies have been affinity-purified and show minimum crossreactivity to bovine, chicken, goat, guinea pig, hamster, horse, human, mouse, rat, and sheep serum proteins. Cross-adsorption or pre-adsorption is a purification step to increase specificity of the antibody resulting in higher sensitivity and less background staining. The secondary antibody solution is passed through a column matrix containing immobilized serum proteins from potentially cross-reactive species. Only the nonspecific-binding secondary antibodies are captured in the column, and the highly specific secondaries flow through. The benefits of this extra step are apparent in multiplexing/multicolor-staining experiments (e.g. flow cytometry) where there is potential cross-reactivity with other primary antibodies or in tissue/cell fluorescent staining experiments where there may be the presence of endogenous immunoglobulins. https://

www.thermofisher.com/antibody/product/Donkey-anti-Rabbit-IgG-H-L-Highly-Cross-Adsorbed-Secondary-Antibody-Polyclonal/A-21207
- Anti-PSAT1 and anti-PARP1 were obtained from the DSHB. Anti-PSAT1 antibody was deposited by Clinical Proteomics Technologies for Cancer at the National Cancer Institute. This antibody has been validated by the Human Protein Atlas. http://www.proteinatlas.org/ENSG000 00135069?PSAT1/antibody.
Anti-PARP1 antibody was deposited by the EU Program Affinomics at the EMBL MACF and was characterized before: https://cordis.europa.eu/project/id/241481/reporting.

Eukaryotic cell lines

Policy information about cell lines

Cell line source(s)	A2780wt and A2780cis cell lines were purchased from ECACC (Sigma Aldrich ECACC 93112519 and Sigma Aldrich ECACC 93112517, respectively). The HEK293T and OVCAR3 cell lines were purchased from ATCC (ATCC CRL-3216™ and NIH:OVCAR-3 ATCC HTB-161™, respectively) .
Authentication	A2780wt and cis cells and OVCAR3 cells were authenticated by the ECACC STR profiling service. The HEK293T cell line was not authenticated by STR profiling because this cell line was only used for virus production.
Mycoplasma contamination	Mycoplasma tests were performed regularly and we confirm that all cell lines tested negative for Mycoplasma (MycoAlert Mycoplasma Detection Kit, Lonza/Westurg).
Commonly misidentified lines (See ICLAC register)	No commonly misidentified cell lines were used.

Animals and other organisms

Policy information about studies involving animals; ARRIVE guidelines recommended for reporting animal research

Laboratory animals	Immunodeficient nude female mice, NMRI-Fox1nu strain (Taconic), were used for establishment and treatment of patient-derived xenografts. Mice were housed in groups of 5 in filter top cages and maintained in a semi-SPF facility at 22°C (+/-2°C) with 14 hours light/10 hours dark cycle and humidity between 45-70%, food and water ad libitum. Bedding was replaced once a week. Mice were 5-10 weeks old at the moment of tumor implantation.
Wild animals	No wild animals were used in this study.
Field-collected samples	No field collected samples were used in this study.
Ethics oversight	The establishment of patient-derived xenografts has been approved by the Commission of Medical Ethics of the University Hospitals Leuven (S54185; ML8713) and by KU Leuven EC (P038/2015).

Note that full information on the approval of the study protocol must also be provided in the manuscript.

Human research participants

Policy information about studies involving human research participants

Population characteristics	Oslo cohort: Immunohistochemistry studies were performed on 18 high-grade serous ovarian cancer effusions from 9 patients (9 primary and 9 recurrent samples). Effusions consisted of 18 HGSC specimens (16 peritoneal, 2 pleural) submitted to the Department of Pathology at the Norwegian Radium Hospital during the period of 1999 to 2008. Clinical information such as age and FIFO staging is indicated in Supplementary table 1 for each patient. Effusion specimens were diagnosed by an experienced cytopathologist based on morphology in smears and cell blocks, prepared using the thrombin clot protocol, and IHC, based on established guidelines. All patient underwent primary debulking surgery before chemotherapy, and for all of them first line chemotherapy consisted out of carboplatin-based treatment. Recurrent biopsies were obtained at late stage disease.<br><br>For the Dutch cohort, IHC analysis was perfomed on 25 pairs of primary and recurrent high-grade serous ovarian cancer tumor samples. Patients with HGSOC FIGO stage IIb-IV, treated with cytoreductive surgery and adjuvant chemotherapy (PDS) or neoadjuvant chemotherapy (NACT) followed by interval debulking surgery in the tertiary referral hospital Netherlands Cancer Institute - Antoni van Leeuwenhoek Hospital (NKI-AVL) between January 2008 and December 2015 were selected. Other covariates such as age, FIGo staging and the presence of metastasis is indicated in Supplementary table 2 for each patient. First line treatment consisted of carboplatin based chemotherapy in 24 patients and other chemotherapy in 1. Recurrence data of the aforementioned patients who were treated in the NKI-AVL were retrieved from patient files. Last check of patient files took place on October 31, 2020. Patients from whom recurrence tissue was extracted, either via biopsy or during surgery, in the NKI-AVL were included. Recurrence samples were obtained both at early and late stage disease. Formalin-fixed, paraffin embedded (FFPE) tissue blocks from both the primary tumor and the recurrence samples were obtained. Diagnosis of the primary tumor was confirmed based on conventional morphological examination of sections stained with hematoxylin and eosin staining. PHGDH IHC was subsequently performed as described in the methods.
Recruitment	No recruitment involved.
Ethics oversight	For the Norwegian study cohort informed consent was obtained according to national and institutional guidelines. Study approval was given by the Regional Committee for Medical Research Ethics in Norway (S-04300).<br>For the Dutch study cohort approval of the institutional review boards of the Netherlands Cancer Registry (NCR, K19.074),

Dutch Pathology Registry (PALGA, 2019-169) and NKI-AVL (CFMPB297) was obtained. The requested dataset was considered anonymous and the use is therefore exempt from ethics review board approval according to Dutch legislation.

Note that full information on the approval of the study protocol must also be provided in the manuscript.

Flow Cytometry

Plots

Confirm that:

☒ The axis labels state the marker and fluorochrome used (e.g. CD4-FITC).

☒ The axis scales are clearly visible. Include numbers along axes only for bottom left plot of group (a 'group' is an analysis of identical markers).

☒ All plots are contour plots with outliers or pseudocolor plots.

☒ A numerical value for number of cells or percentage (with statistics) is provided.

Methodology

Sample preparation	Tumor cell lines were incubated with appropriate fluorescent probes (EdU Alexa Fluor 488, FX Cycle Violet, MitoSOX or CellROX deep red) according to the manufacturer's protocol. Next cells were dissociated by 0.25% trypsin-EDTA, centrifuged, washed, centrifuged, dissolved in PBS, filtered over a cell strainer and fluorescence was measured.
Instrument	BD FACSCanto II, BD Biosciences
Software	data collection: BD FACS Diva ; data analysis: FlowJo Version 10.7.1
Cell population abundance	10 000 events per sample reading were recorded. The gating strategy selected single, live cells. For control A2780 wt and cis cells, this population made up ~90% of the total starting populations. Upon carboplatin treatment or ser/gly deprivation the population dropped to ~20-50% depending on the specific condition.
Gating strategy	Singlets, live cells were gated using FSC/SSC gating.<br>For MitoSOX and CellROX staining, levels of positivity was determined by the specific molecular probes, and measurements were distinct from negative controls. Gating example is represented in Supplementary Figure 3c,d.<br>For cell cycle analysis, live cells were dual stained with FXCycle and EdU probes, enabling division of cells depending of cell cycle stage with gating represented in supplementary figure 7c.

☒ Tick this box to confirm that a figure exemplifying the gating strategy is provided in the Supplementary Information.
